# Supplementary material for: Loss of cell-autonomously secreted laminin-α2 drives muscle stem cell dysfunction in LAMA2-related muscular dystrophy
Source: Nat Commun. 2025 Nov 27;16:10674. doi: 10.1038/s41467-025-65703-1 (PMC12661008; doi:10.1038/s41467-025-65703-1)
Supplement: Supplementary file 1 — Supplementary Information [file 41467_2025_65703_MOESM1_ESM.pdf]

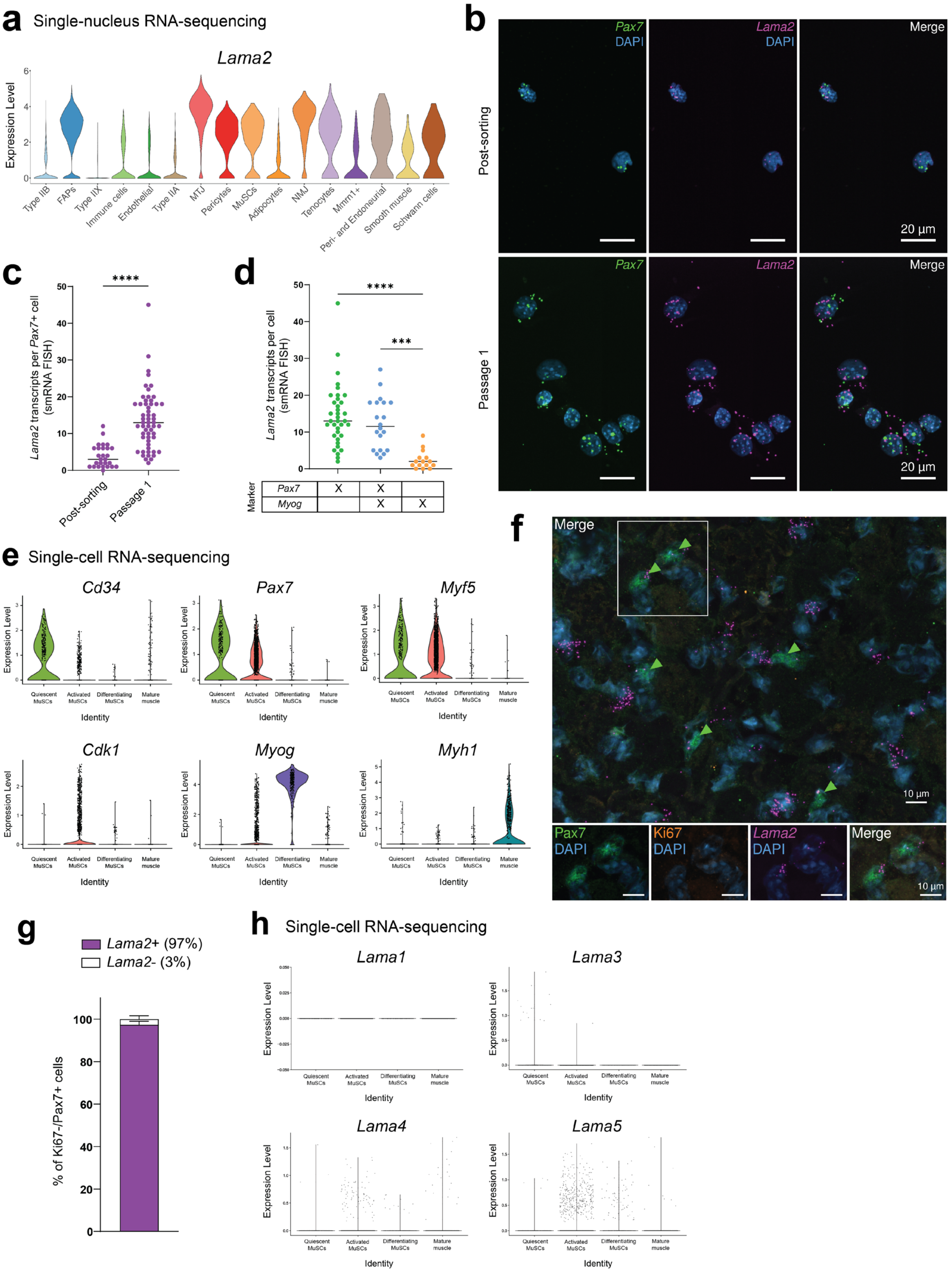
a
Single-nucleus RNA-sequencing
Lama2
Expression Level
Type IIB
FAPs
Type IIX
Immune cells
Endothelial
Type IIA
MTJ
Pericytes
MuSCs
Adipocytes
NMJ
Tenocytes
Mmrn1+
Peri- and Endoneurial
Smooth muscle
Schwann cells
b
Pax7
DAPI
Lama2
DAPI
Merge
Post-sorting
Passage 1
20 µm
c
****
Lama2 transcripts per Pax7+ cell (smRNA FISH)
Post-sorting
Passage 1
d
****
***
Lama2 transcripts per cell (smRNA FISH)
Marker
Pax7
Myog
X
e
Single-cell RNA-sequencing
Cd34
Pax7
Myf5
Cdk1
Myog
Myh1
Expression Level
Quiescent MuSCs
Activated MuSCs
Differentiating MuSCs
Mature muscle
Identity
f
Merge
10 µm
Pax7
DAPI
Ki67
DAPI
Lama2
DAPI
Merge
g
Lama2+ (97%)
Lama2- (3%)
% of Ki67-/Pax7+ cells
h
Single-cell RNA-sequencing
Lama1
Lama3
Lama4
Lama5
Expression Level
Quiescent MuSCs
Activated MuSCs
Differentiating MuSCs
Mature muscle
Identity

Fig. S1: Quiescent and activated MuSCs express *Lama2*. **a** Violin plot showing *Lama2* expression in previously-defined cell types of *tibialis anterior* (TA) and *gastrocnemius* muscles of adult C57BL/6 mice[1]. **b** Representative smRNA FISH in primary myoblasts immediately post-sorting (1st row) or after the first passage (2nd row) (*Pax7* in green, *Lama2* in magenta, DAPI in blue). **c** Quantification of *Lama2* transcripts detected by smRNA FISH in *Pax7*+ cells immediately post-sorting or after the first passage. Each dot represents one cell; cells were isolated from n = 2 mice. **d** Quantification of the number of *Lama2* transcripts detected by smRNA FISH in *Pax7*+/*Myog*-, *Pax7*+/*Myog*+ and *Pax7*-/*Myog*+ cells after the first passage. Each dot represents one cell; cells were isolated from n = 2 mice. **e** Violin plots showing the expression of markers used to sub-cluster MuSCs in a single-cell RNA-sequencing dataset generated from injured and uninjured TA muscles of adult C57BL/6 mice[2]. **f** Representative immunostaining and smRNA FISH in uninjured TA of adult C57BL/6 mice (Pax7 in green, Ki67 in orange, *Lama2* in magenta, DAPI in blue). Green arrows indicate Ki67-/Pax7+ cells. **g** Quantification of the proportion of quiescent Ki67-/Pax7+ MuSCs co-localizing with *Lama2* in uninjured TA of adult C57BL/6 mice (n = 7 mice). **h** Violin plots showing the expression of *Lama* genes in the single-cell RNA-sequencing dataset[2]. Statistical significance was determined by unpaired student's two-sided t-test (**c**) or one-way ANOVA with Tukey's multiple comparisons test (**d**). ***P < 0.001; ****P < 0.0001. Source data and P-values are provided as a Source Data file.

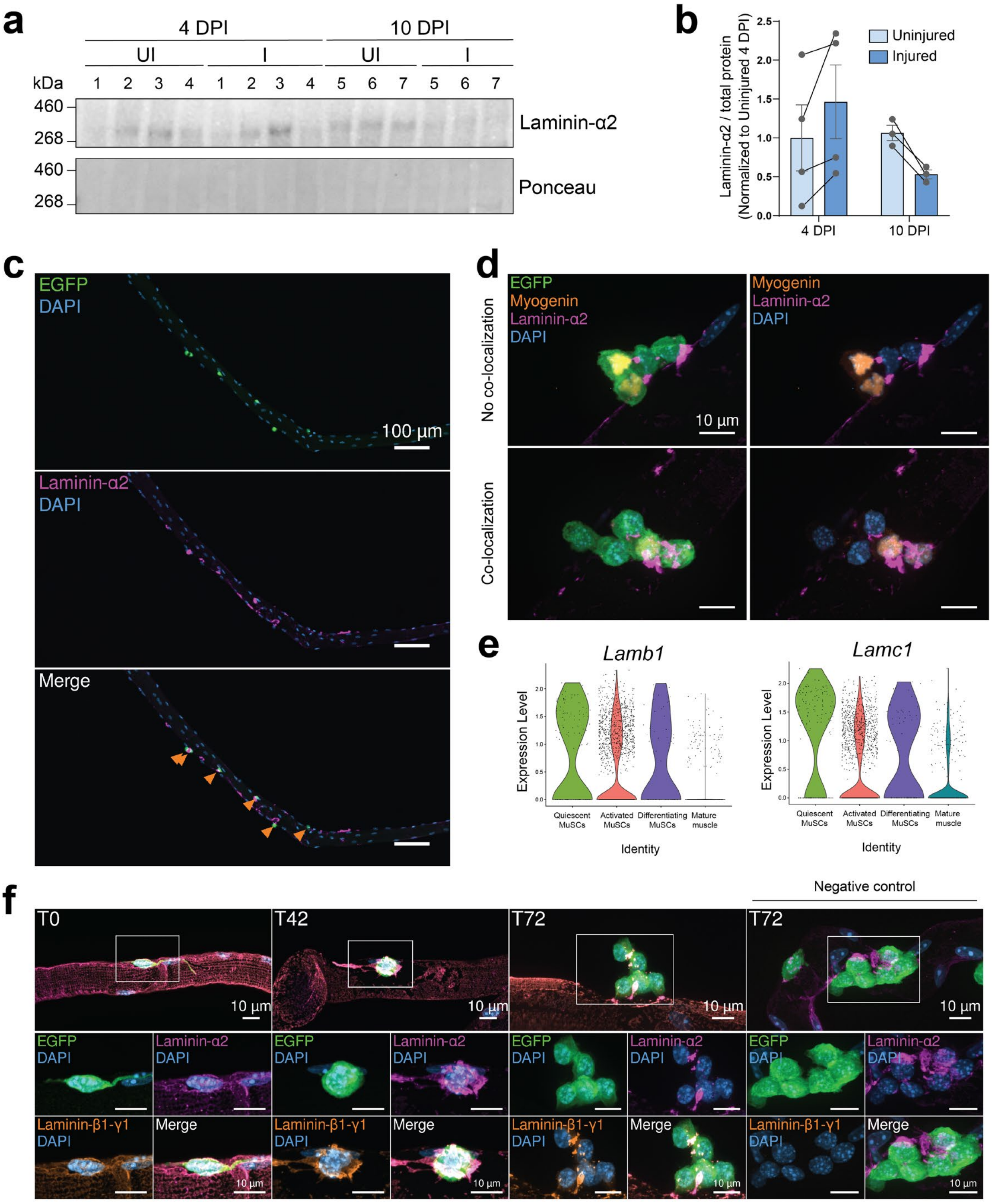

a
4 DPI
10 DPI
UI
I
kDa
1 2 3 4 1 2 3 4 5 6 7 5 6 7
460
268
Laminin-α2
Ponceau
b
Laminin-α2 / total protein (Normalized to Uninjured 4 DPI)
Uninjured
Injured
4 DPI
10 DPI
c
EGFP
DAPI
100 µm
Laminin-α2
Merge
d
No co-localization
Co-localization
Myogenin
10 µm
e
Lamb1
Lamc1
Expression Level
Quiescent MuSCs
Activated MuSCs
Differentiating MuSCs
Mature muscle
Identity
f
T0
T42
T72
Negative control
10 µm
Laminin-β1-γ1

Fig. S2: Activated MuSCs remodel their microenvironment with laminin-211. a Western blot analysis for laminin-α2 and total protein stains (Ponceau) in injured (I) and uninjured contralateral (UI) TAs at 4 and 10 days post-injury (DPI). Lane numbers indicate TAs from the same mouse. **b** Quantification of laminin-α2 abundance in injured and uninjured TAs at 4 and 10 DPI (normalized to total protein stain). Lines show injured and contralateral uninjured TAs from the same mouse. *n* = 4 mice at 4 DPI; *n* = 3 mice at 10 DPI. **c** Immunostaining of an EDL fiber with EGFP-labelled MuSCs after 42 h in culture (EGFP in green, laminin-α2 in magenta, DAPI in blue). Orange arrows in the merge panel indicate EGFP+ MuSCs. This experiment was repeated twice in *n* = 4 mice with similar results. **d** Immunostainings of EDL fibers with EGFP-labelled MuSCs at T72 showing no co-localization (1st row) and co-localization (2nd row) of Myogenin+/EGFP+ cells with laminin-α2 (EGFP in green, Myogenin in orange, laminin-α2 in magenta, DAPI in blue). This experiment was performed once in *n* = 4 mice with similar results. **e** Violin plots showing the expression of *Lamb1* and *Lamc1* in a single-cell RNA-sequencing dataset[2]. **f** Immunostaining of EDL fibers with EGFP-labelled MuSCs after 0 (T0), 42 (T42) and 72 (T72) hours in culture (EGFP in green, laminin-α2 in magenta, laminin-β1-γ1 in orange, DAPI in blue). For the negative control (panel on the far right), the anti-laminin-β1-γ1 primary antibody was omitted. This experiment was performed once in *n* = 4 mice with similar results. In **b,** statistical significance was determined by paired student's two-sided t-test. Source data and *P*-values are provided as a Source Data file.

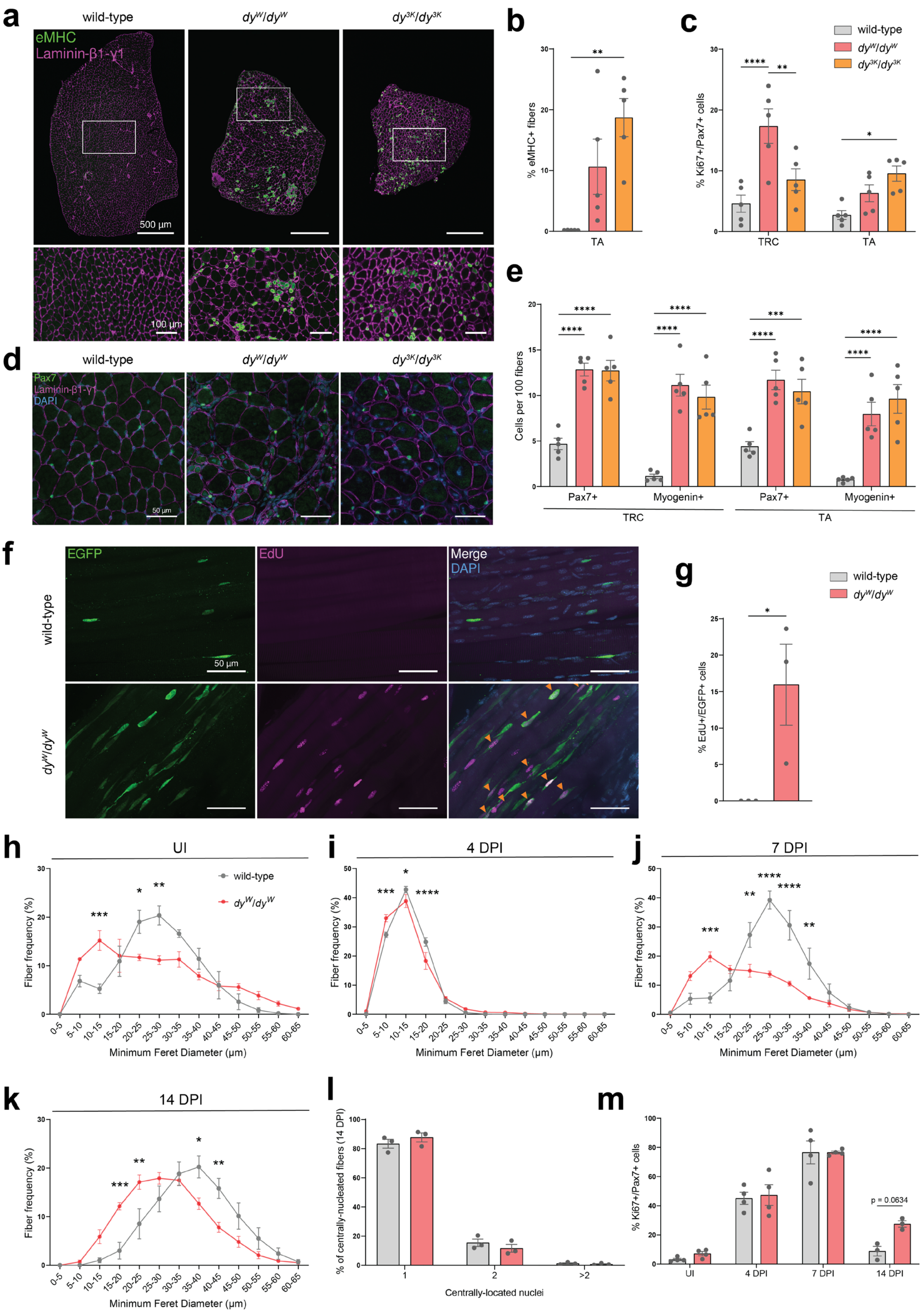

a
wild-type
dyW/dyW
dy3K/dy3K
eMHC
Laminin-β1-γ1
500 µm
100 µm
b
% eMHC+ fibers
TA
c
% Ki67+/Pax7+ cells
TRC
TA
wild-type
dyW/dyW
dy3K/dy3K
d
Pax7
Laminin-β1-γ1
DAPI
50 µm
e
Cells per 100 fibers
Pax7+
Myogenin+
TRC
TA
f
EGFP
EdU
Merge
DAPI
50 µm
g
% EdU+/EGFP+ cells
h
UI
Fiber frequency (%)
Minimum Feret Diameter (µm)
i
4 DPI
j
7 DPI
k
14 DPI
l
% of centrally-nucleated fibers (14 DPI)
Centrally-located nuclei
m
% Ki67+/Pax7+ cells
UI
4 DPI
7 DPI
14 DPI
p = 0.0634

Fig. S3: LAMA2 MD mouse models have high levels of endogenous muscle repair. a Representative immunostainings of TA cross-sections of wild-type, dy^{W}/dy^{W} and dy^{3K}/dy^{3K} mice at 4 weeks of age with antibodies to embryonic myosin heavy chain (eMHC), depicting recently regenerated muscle fibers (eMHC in green, laminin-β1-γ1 in magenta). **b** Quantification of the proportion of eMHC+ fibers at 4 weeks of age. *n* = 5 mice. **c** Quantification of the proportion of Ki67+/Pax7+ cells at 4 weeks of age. *n* = 5 mice. **d** Representative immunostaining of Pax7+ MuSCs in the TRC at 4 weeks of age (Pax7 in green, laminin-β1-γ1 in magenta, DAPI in blue). **e** Quantification of the number of Pax7+ and Myogenin+ cells per 100 fibers in the TRC and TA. *n* = 5 mice. **f** Whole-mount immunostaining of EGFP-labelled MuSCs in the EDL after a 24 h EdU chase (EGFP in green, EdU in magenta, DAPI in blue). EdU+/EGFP+ cells are indicated by orange arrowheads in the merge panel. **g** Quantification of EdU+/EGFP+ MuSCs in EDL whole-mounts. *n* = 3 mice. **h** – **k** Fiber size distribution in uninjured (UI) conditions (**h**), and at four (**i**), seven (**j**) and fourteen (**k**) days post-injury (DPI). *n* = 3 mice. **l** Quantification of the number of centrally-located nuclei in centrally-nucleated fibers at 14 DPI. *n* = 3 mice. **m** Quantification of the proportion of Ki67+/Pax7+ cells post-injury. *n* = 4 mice at 4 and 7 DPI; *n* = 3 mice at 14 DPI. Data are means ± SEM. Statistical significance was determined by one-way ANOVA with Tukey's multiple comparisons test (**b**) or two-way ANOVAs with Tukey's multiple comparisons test (**c** and **e**) or two-way ANOVAs with Bonferroni's multiple comparisons test (**h**, **i**, **j**, **k**, and **m**) or unpaired student's two-sided t-test (**g** and **l**). $^{*}P < 0.05$; $^{**}P < 0.01$; $^{***}P < 0.001$; $^{****}P < 0.0001$. Source data and *P*-values are provided as a Source Data file.

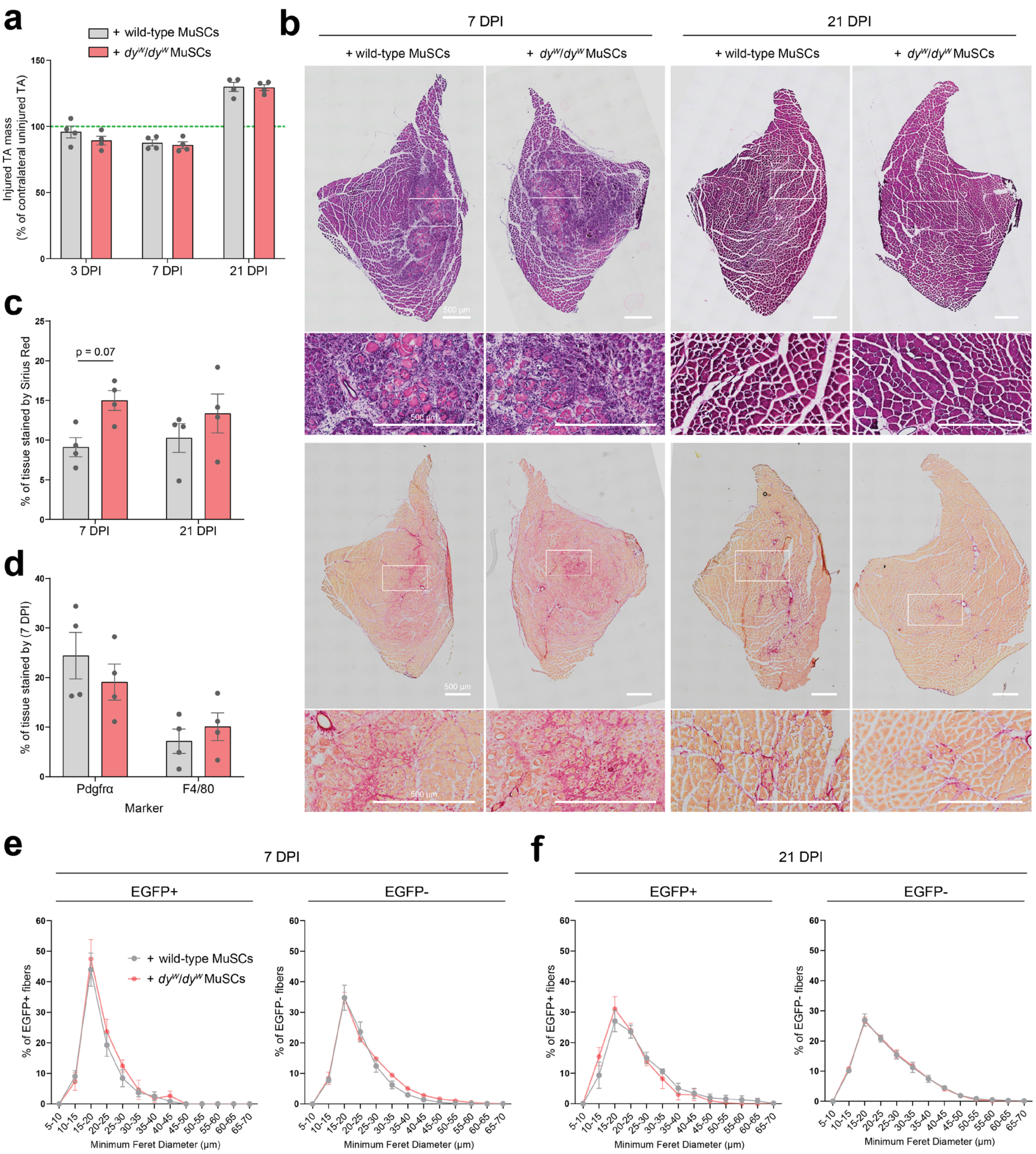
a
+ wild-type MuSCs
+ dy^W/dy^W MuSCs
Injured TA mass (% of contralateral uninjured TA)
3 DPI
7 DPI
21 DPI
b
7 DPI
21 DPI
+ wild-type MuSCs
+ dy^W/dy^W MuSCs
500 µm
c
% of tissue stained by Sirius Red
p = 0.07
d
% of tissue stained by (7 DPI)
Pdgfrα
F4/80
Marker
e
f
EGFP+
EGFP-
% of EGFP+ fibers
% of EGFP- fibers
Minimum Feret Diameter (µm)

Fig. S4: Transplanted *dy^W/dy^W* MuSCs do not impair regeneration. a Quantification of TA masses at 3, 7 and 21 days post-injury (DPI). Injured TA masses are normalized to the contralateral uninjured TA's mass (green dotted line). *n* = 4 mice. **b** Representative H&E (top) and Picro-Sirius Red (bottom) stains of recipient TA cross-sections at 7 (left) and 21 (right) DPI. **c** Quantification of the proportion of tissue stained by Sirius Red at 7 and 21 DPI in TA cross-sections. *n* = 4 mice. **d** Quantification of the proportion of tissue stained by Pdgfrα and F4/80 at 7 DPI in TA cross-sections. *n* = 4 mice. **e** Fiber size distribution of EGFP+ (left) and EGFP- (right) fibers at 7 DPI. *n* = 4 mice. **f** Fiber size distribution of EGFP+ (left) and EGFP- (right) fibers at 21 DPI. *n* = 4 mice. Data are means ± SEM. Statistical significance was determined by two-way ANOVAs with Bonferroni's multiple comparisons test (**a**, **c**, **e**, and **f**) or unpaired student's two-sided t-test (**d**). Source data and *P*-values are provided as a Source Data file.

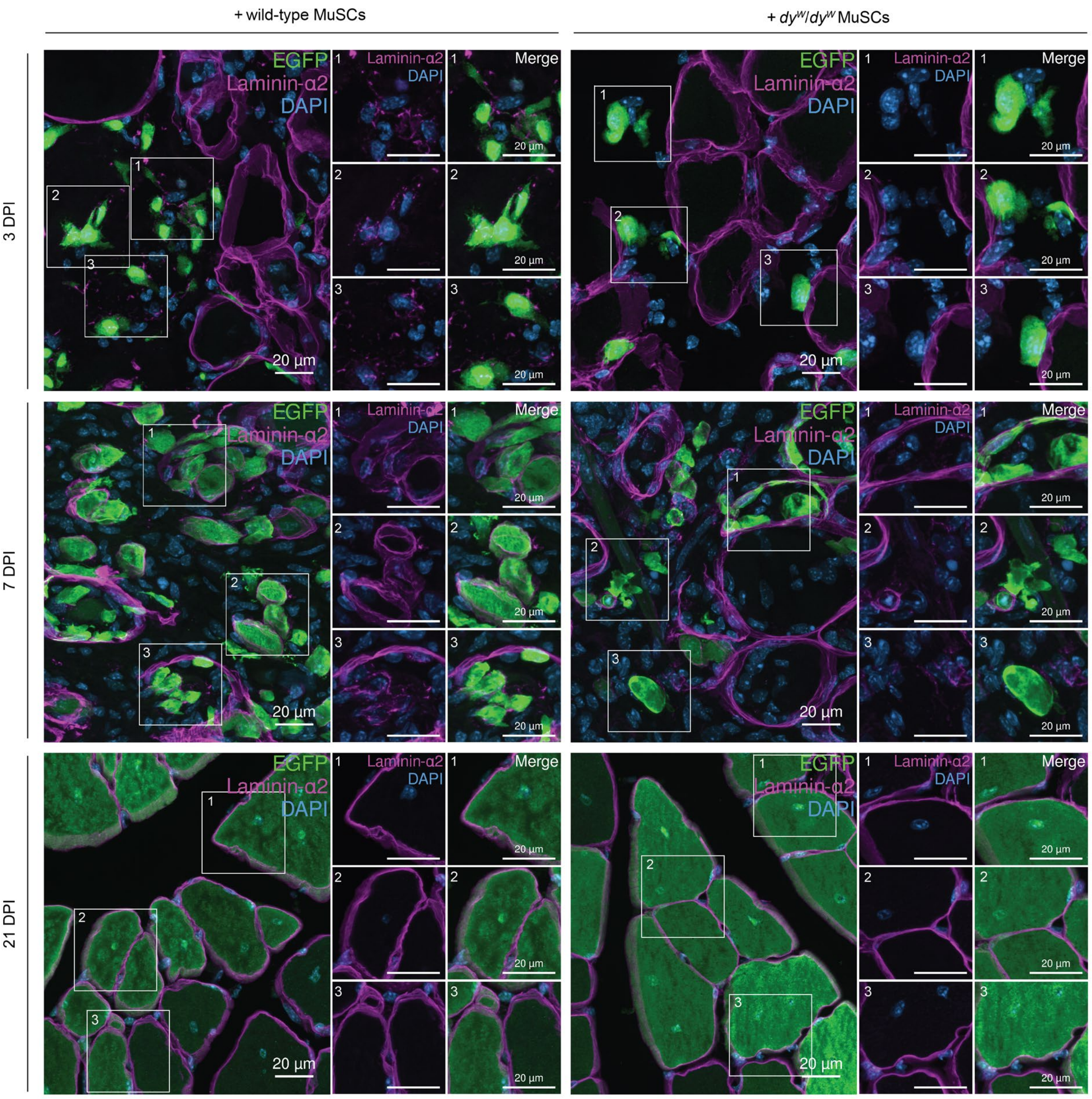

+ wild-type MuSCs
+ dy^W/dy^W MuSCs
3 DPI
7 DPI
21 DPI
EGFP
Laminin-α2
DAPI
Merge
20 µm
1
2
3

Fig. S5: Proliferating wild-type MuSCs co-localize with laminin-α2 post-transplantation. Representative images of recipient TA cross-sections at 3, 7 and 21 days post-injury (DPI) (EGFP in green, laminin-α2 in magenta, DAPI in blue). For each image, three zoomed-in panels are shown. At 3 DPI, laminin-α2 co-localizes with transplanted EGFP+ wild-type MuSCs, but not with transplanted EGFP+ dy^W/dy^W MuSCs. At 7 DPI, laminin-α2 is occasionally in the vicinity of dy^W/dy^W MuSCs, but at much lower levels than around wild-type MuSCs. At 21 DPI, all EGFP+ fibers are similarly surrounded by laminin-α2. This experiment was performed once in $n = 4$ mice per time point with similar results.

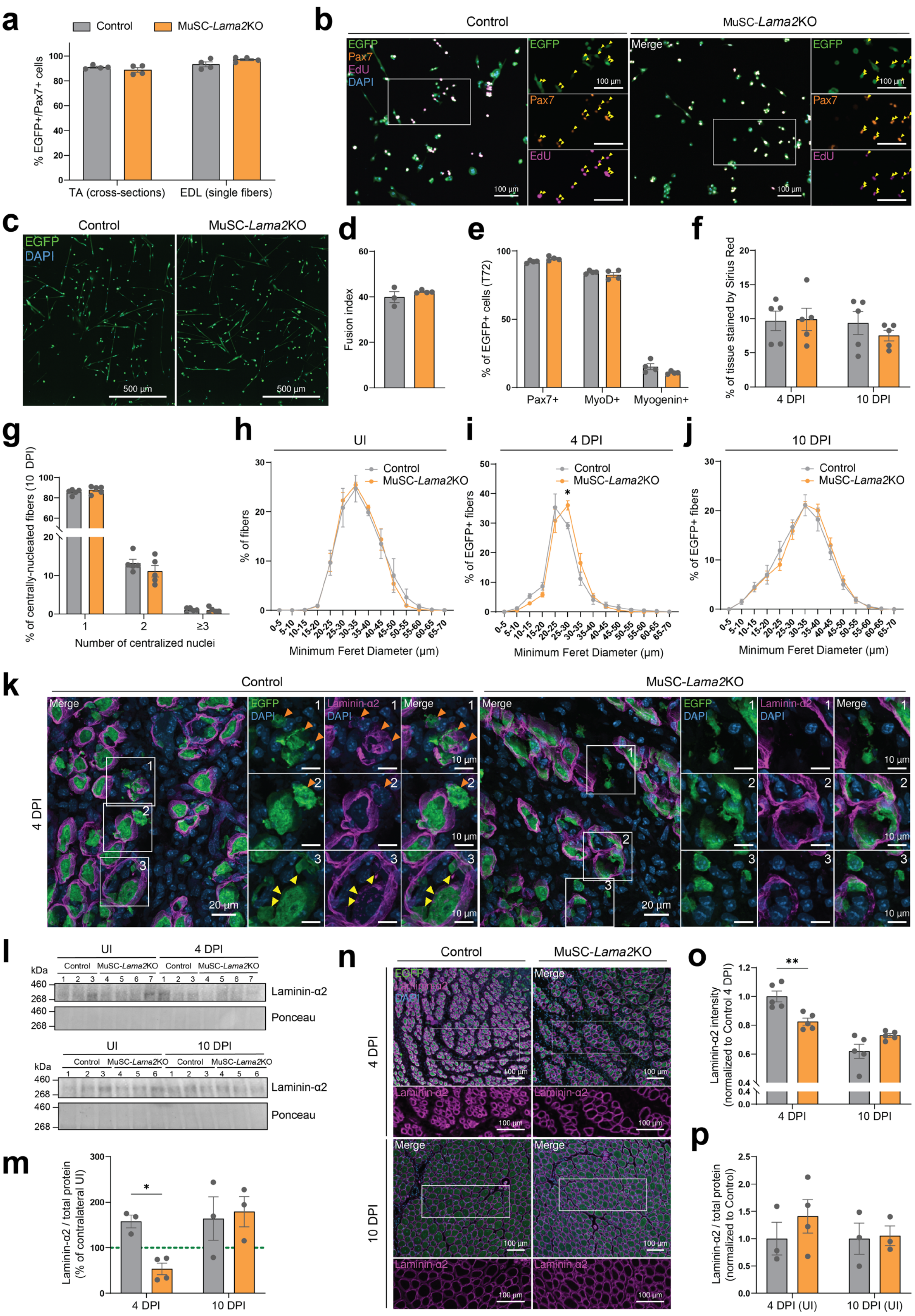
a
Control
MuSC-Lama2KO
% EGFP+/Pax7+ cells
TA (cross-sections)
EDL (single fibers)
b
Control
MuSC-Lama2KO
EGFP
Pax7
EdU
DAPI
Merge
100 µm
c
Control
MuSC-Lama2KO
EGFP
DAPI
500 µm
d
Fusion index
e
% of EGFP+ cells (T72)
Pax7+
MyoD+
Myogenin+
f
% of tissue stained by Sirius Red
4 DPI
10 DPI
g
% of centrally-nucleated fibers (10 DPI)
Number of centralized nuclei
1
2
≥3
h
UI
Control
MuSC-Lama2KO
% of fibers
Minimum Feret Diameter (µm)
i
4 DPI
% of EGFP+ fibers
j
10 DPI
k
Control
MuSC-Lama2KO
4 DPI
Merge
EGFP
DAPI
Laminin-α2
20 µm
10 µm
l
UI
4 DPI
10 DPI
Control
MuSC-Lama2KO
kDa
460
268
Laminin-α2
Ponceau
m
Laminin-α2 / total protein (% of contralateral UI)
4 DPI
10 DPI
n
Control
MuSC-Lama2KO
4 DPI
10 DPI
EGFP
Laminin-α2
DAPI
Merge
100 µm
o
Laminin-α2 intensity (normalized to Control 4 DPI)
4 DPI
10 DPI
p
Laminin-α2 / total protein (normalized to Control)
4 DPI (UI)
10 DPI (UI)

Fig. S6: MuSC-specific *Lama2* knockout is sufficient to impair MuSC proliferation and delay regeneration. a Quantification of the proportion of EGFP+/Pax7+ cells in TA cross-sections and on single fibers isolated from the EDL after 5 consecutive days of tamoxifen treatment. *n* = 4 mice. **b** Representative immunostaining of cultured primary myoblasts after EdU incubation from 6-21 h post-plating (EGFP in green, Pax7 in orange, EdU in magenta, DAPI in blue). Yellow arrowheads: EdU+/Pax7+/EGFP+ cells. **c** Representative immunostaining of tissue culture dishes after 3 days in differentiation medium (EGFP in green, DAPI in blue). **d** Quantification of the fusion index (number of nuclei residing in myotubes divided by the total number of nuclei) after 3 days in differentiation medium. *n* = 3 mice for control; *n* = 4 mice for MuSC-*Lama2*KO. **e** Quantification of the proportion of Pax7+, MyoD+ and Myogenin+ EGFP+ cells on single EDL fibers at T72. *n* = 4 mice; with a minimum of 20 fibers analyzed per mouse. **f** Quantification of the proportion of tissue stained by Sirius Red at 4 and 10 DPI in TA cross-sections. *n* = 5 mice. **g** Quantification of the number of centrally-located nuclei at 10 DPI. *n* = 5 mice. **h** – **j** Fiber size distribution in uninjured (UI) conditions (**h**) and four (**i**) and ten days (**j**) post-cardiotoxin injury. In **h**, *n* = 4 mice; in **i** and **j**, *n* = 5 mice. **k** Representative immunostaining of TA cross-sections from control and MuSC-*Lama2*KO mice at 4 DPI. Orange arrows: laminin-α2 co-localization with interstitial EGFP+ cells; yellow arrows: laminin-α2 presence on the apical side of EGFP+ cells in ghost fibers. **l** Western blot analysis for laminin-α2 and total protein stains (Ponceau) in injured and contralateral uninjured (UI) TAs at 4 (top) and 10 DPI (bottom). Lane numbers indicate TAs from the same mouse. **m** Quantification of laminin-α2 abundance at 4 and 10 DPI (normalized to total protein stain and shown as a percentage of contralateral uninjured TA). At 4 DPI, *n* = 3 control mice; *n* = 4 MuSC-*Lama2*KO mice. At 10 DPI, *n* = 4 mice. **n** Representative laminin-α2 immunostaining in TA cross-sections at 4 and 10 DPI (EGFP in green, laminin-α2 in magenta, DAPI in blue). **o** Quantification of laminin-α2 signal intensity in TA cross-sections at 4 and 10 DPI (average of 2 sections per mouse; *n* = 5 mice). **p** Quantification of laminin-α2 abundance in uninjured TAs at 4 and 10 DPI by Western blot (normalized to total protein and subsequently normalized to controls). At 4 DPI, *n* = 3 control mice; *n* = 4 MuSC-*Lama2*KO mice. At 10 DPI, *n* = 3 mice. Data are means ± SEM. Statistical significance was determined by unpaired student's two-sided t-test (**a**, **d**, **e**, and **g**) or two-way ANOVAs with Bonferroni's multiple comparisons test (**f**, **h**, **i**, **j**, **m**, **o**, and **p**). *$P < 0.05$; **$P < 0.01$. Source data and *P*-values are provided as a Source Data file.

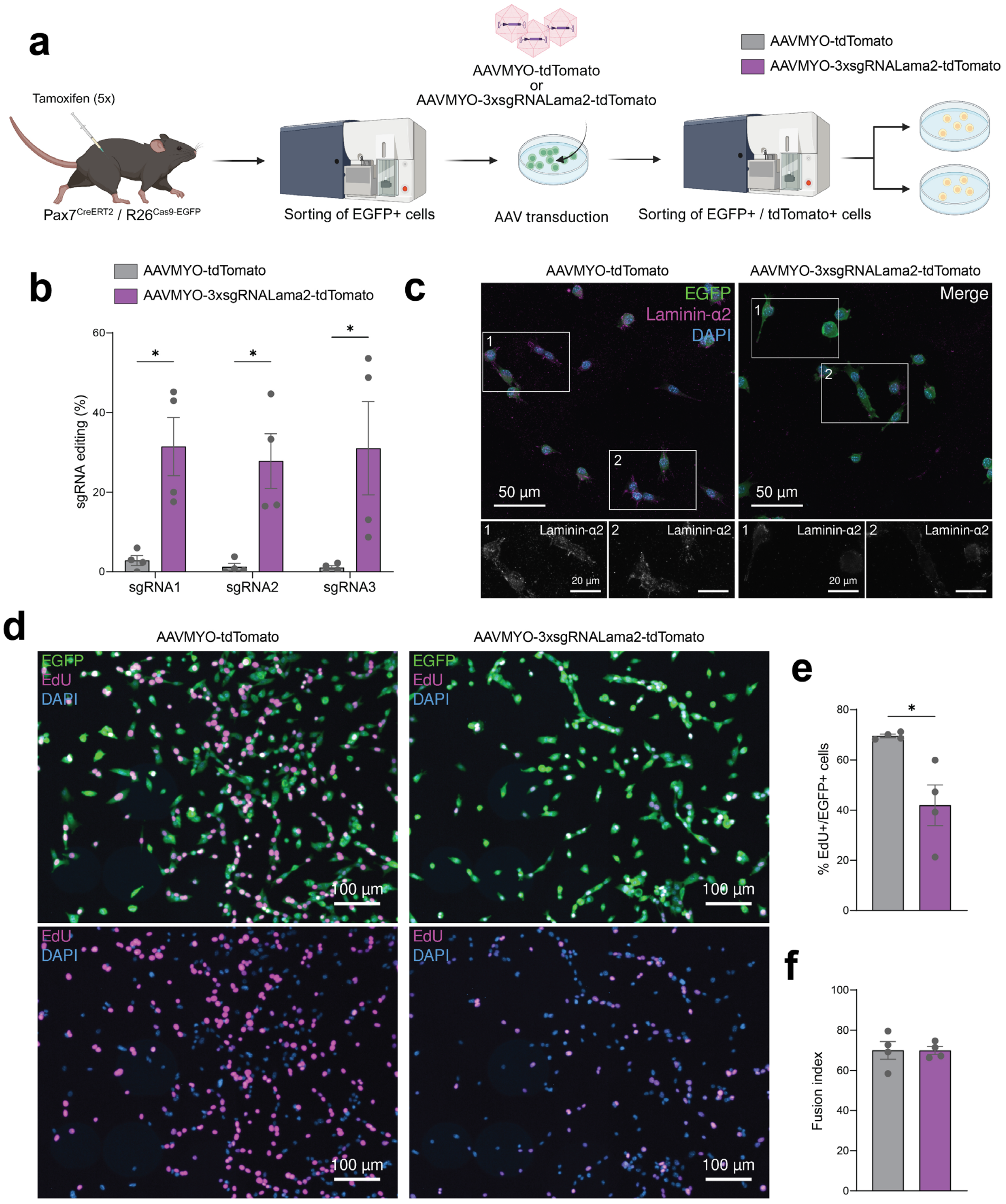
a
Tamoxifen (5x)
Pax7CreERT2 / R26Cas9-EGFP
Sorting of EGFP+ cells
AAVMYO-tdTomato
or
AAVMYO-3xsgRNALama2-tdTomato
AAV transduction
Sorting of EGFP+ / tdTomato+ cells
AAVMYO-tdTomato
AAVMYO-3xsgRNALama2-tdTomato
b
AAVMYO-tdTomato
AAVMYO-3xsgRNALama2-tdTomato
sgRNA editing (%)
sgRNA1
sgRNA2
sgRNA3
c
AAVMYO-tdTomato
AAVMYO-3xsgRNALama2-tdTomato
EGFP
Laminin-α2
DAPI
Merge
50 µm
20 µm
d
AAVMYO-tdTomato
AAVMYO-3xsgRNALama2-tdTomato
EGFP
EdU
DAPI
100 µm
e
% EdU+/EGFP+ cells
f
Fusion index

Fig. S7: CRISPR/Cas9-mediated *Lama2* knockout reduces MuSC proliferation *ex vivo*.
a Experimental approach: mice with inducible MuSC-specific expression of Cas9 and EGFP[3, 4] were treated with tamoxifen for 5 consecutive days. EGFP+ cells were then isolated *via* FACS, expanded on collagen-coated tissue culture dishes before AAVMYO was added to medium to deliver either a CMV-driven tdTomato transgene (AAVMYO-tdTomato), or a CMV-driven tdTomato transgene and 3 U6-driven single guide RNAs (sgRNAs) targeting exons 2 and 3 of *Lama2* (AAVMYO-3xsgRNALama2-tdTomato). Cells were passaged four days after the addition of AAVMYO. Transduced EGFP+/tdTomato+ cells were sorted *via* FACS and re-plated to assess the effect of *Lama2* mutations on isogenic primary myoblasts. **b** Quantification of sgRNA editing efficiency by Tracking of Indels by Decomposition (TIDE) (see Methods for more details). **c** Representative immunostaining of laminin-α2 in tissue culture dishes containing isogenic EGFP+ cells that received either AAVMYO-tdTomato or AAVMYO-3xsgRNALama2-tdTomato (EGFP in green, laminin-α2 in magenta, DAPI in blue). Boxes 1 and 2 are zoomed-in panels showing laminin-α2 in white. This experiment was performed once with cells isolated from $n = 4$ mice with similar results. **d** Representative immunostaining of isogenic EGFP+ cells that were transduced with either AAVMYO-tdTomato or AAVMYO-3xsgRNALama2-tdTomato. EdU was added to the medium 2 days after the sorting of EGFP+/tdTomato+ cells, and cells were fixed 15 h later (EGFP in green, EdU in magenta, DAPI in blue). **e** Quantification of the proportion of EdU+/EGFP+ cells after a 15 h incubation with EdU. **f** Quantification of the fusion index (number of nuclei residing in myotubes divided by the total number of nuclei) after 7 days of differentiation. In **b**, **e**, and **f**, cells were isolated from $n = 4$ mice. Data are means ± SEM. In all graphs, statistical significance was determined by unpaired student's two-sided t-test. *$P < 0.05$. Source data and *P*-values are provided as a Source Data file. **a** Created in BioRender. Ruegg, M. (2025) https://BioRender.com/d18khcw.

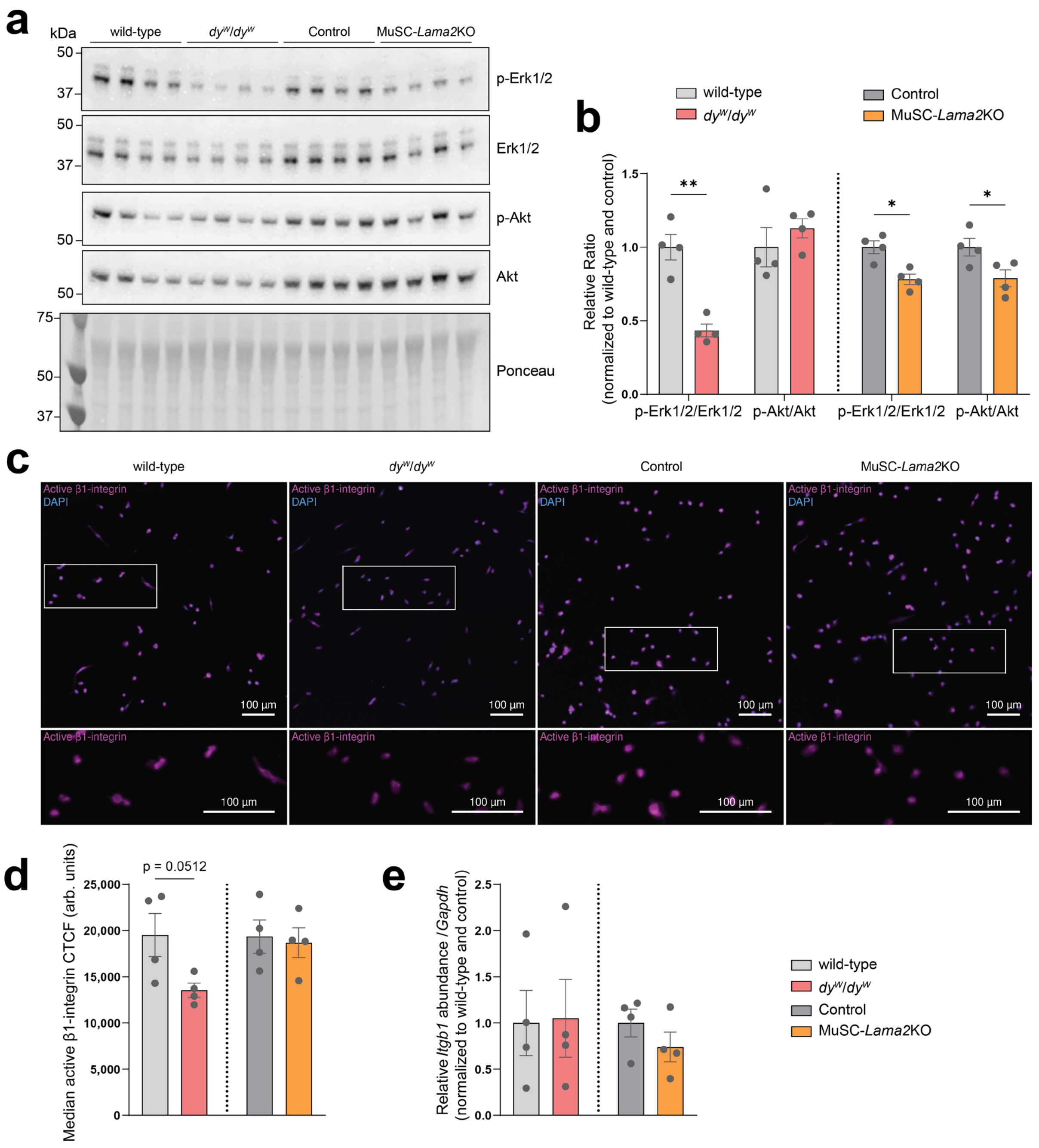
a
kDa
wild-type
dy^W/dy^W
Control
MuSC-Lama2KO
50
37
p-Erk1/2
Erk1/2
p-Akt
Akt
75
Ponceau
b
wild-type
dy^W/dy^W
Control
MuSC-Lama2KO
Relative Ratio (normalized to wild-type and control)
1.5
1.0
0.5
0.0
**
*
p-Erk1/2/Erk1/2
p-Akt/Akt
c
Active β1-integrin
DAPI
100 µm
d
Median active β1-integrin CTCF (arb. units)
p = 0.0512
25,000
20,000
15,000
10,000
5,000
0
e
Relative Itgb1 abundance /Gapdh (normalized to wild-type and control)
2.5
2.0

Fig. S8: Possible mechanisms involved in the proliferative deficits of *Lama2*-deficient MuSCs. a Western blot analysis for phosphorylated Erk1/2 (p-Erk1/2), Erk1/2, phosphorylated Akt (p-Akt) and Akt in wild-type, dy^W/dy^W, control and MuSC-*Lama2*KO primary myoblasts (PMs) *ex vivo.* **b** Quantification of the ratio of p-Erk1/2 to total Erk1/2 and p-Akt to total Akt detected by Western blot. **c** Representative immunostaining of active β1-integrin (9EG7 antibody) in wild-type, dy^W/dy^W, control and MuSC-*Lama2*KO PMs, cultured on collagen-coated tissue culture dishes (active β1-integrin in magenta, DAPI in blue). **d** Quantification of the amount of active β1-integrin (9EG7 antibody) by Corrected Total Cell Fluorescence (CTCF) (>150 cells were quantified from 2 fields of view for each sample). See Methods for details on CTCF calculation. **e** Quantification of relative *Itgb1* mRNA levels in wild-type, dy^W/dy^W, control and MuSC-*Lama2*KO PMs by RT-qPCR. In **b**, **d**, and **e**, cells were isolated from n = 4 mice. Data are means ± SEM. In all graphs, statistical significance was determined by unpaired student's two-sided t-test. *P < 0.05; **P < 0.01. Source data and P-values are provided as a Source Data file.

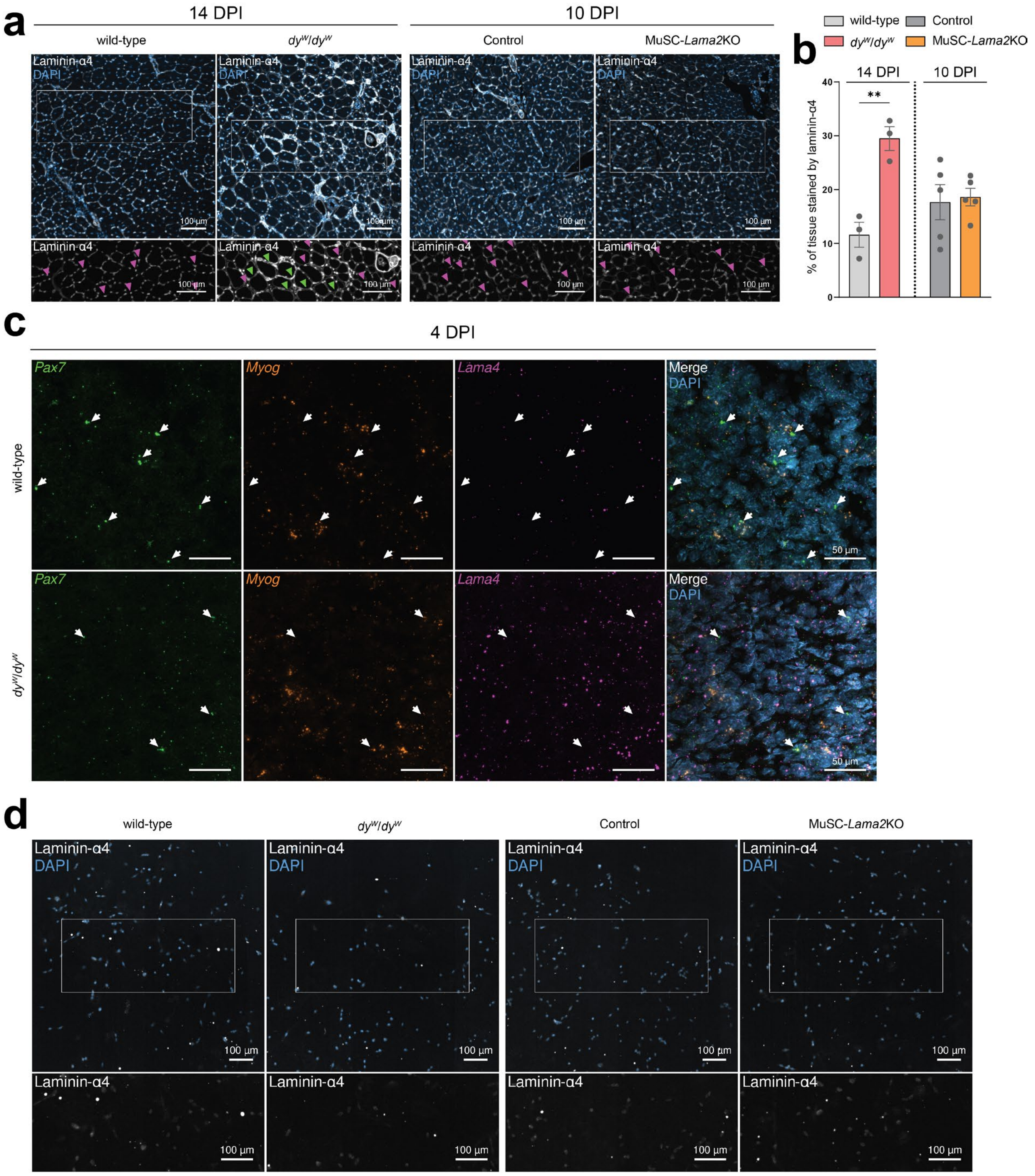
a
14 DPI
10 DPI
wild-type
dyW/dyW
Control
MuSC-Lama2KO
Laminin-α4
DAPI
100 µm
b
wild-type
dyW/dyW
Control
MuSC-Lama2KO
14 DPI
10 DPI
**
% of tissue stained by laminin-α4
0
10
20
30
40
c
4 DPI
Pax7
Myog
Lama4
Merge
DAPI
50 µm
wild-type
dyW/dyW
d
wild-type
dyW/dyW
Control
MuSC-Lama2KO
Laminin-α4
DAPI
100 µm

Fig. S9: *Lama2*-deficient MuSCs do not upregulate laminin-α4. a Representative immunostaining of wild-type and dy^W/dy^W TAs at 14 days post-injury (DPI), and control and MuSC-*Lama2*KO TAs at 10 DPI (laminin-α4 in white, DAPI in blue). In the lower panels, magenta arrows point to the punctate staining of blood vessels and green arrows show laminin-α4 surrounding muscle fibers. **b** Quantification of the proportion of tissue stained by laminin-α4 in TA cross-sections. At 14 DPI, n = 3 mice; at 10 DPI, n = 5 mice. **c** smRNA FISH in wild-type and dy^W/dy^W TAs at 4 DPI (*Pax7* in green, *Myog* in orange, *Lama4* in magenta, DAPI in blue). White arrows indicate *Pax7*+ cells. This experiment was performed once with n = 2 mice with similar results. **d** Representative immunostaining of wild-type, dy^W/dy^W, control and MuSC-*Lama2*KO primary myoblasts cultured in proliferation medium for 5 days on collagen-coated plates (laminin-α4 in white, DAPI in blue). This experiment was performed once with cells isolated from n = 4 mice with similar results. Data are means ± SEM. In **b**, statistical significance was determined by unpaired student's two-sided t-test. $^{**}P < 0.01$. Source data and *P*-values are provided as a Source Data file.

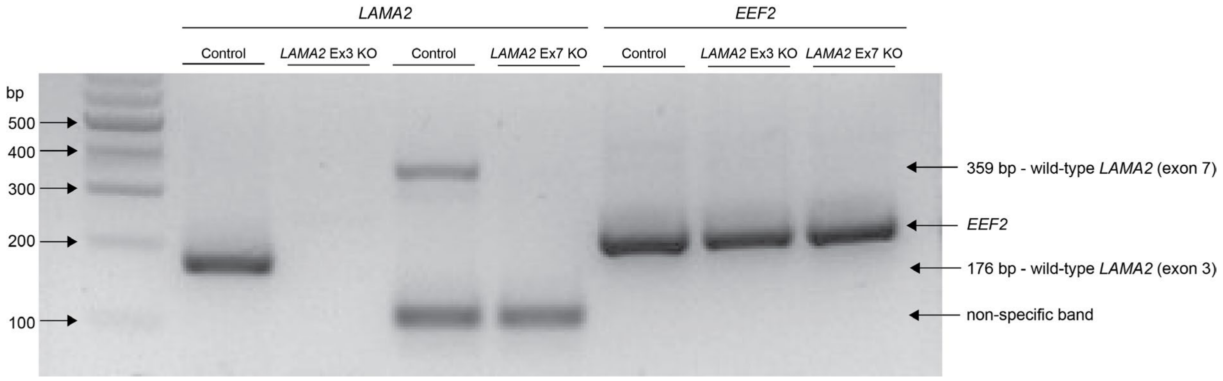

Fig. S10: Engineered hiPSCs do not express full-length *LAMA2* mRNA. RT-PCR showing the loss of full-length *LAMA2* mRNA in *LAMA2* Ex3 KO and *LAMA2* Ex7 KO hiPSCs. The expression of *EEF2* is shown for all samples as a control. In the comparison of *LAMA2* expression in control versus *LAMA2* Ex3 KO cells, the forward primer targets exon 2 and the reverse primer targets exon 3 (product length = 176 bp). In the comparison of *LAMA2* expression in control versus *LAMA2* Ex7 KO cells, the forward primer targets exon 6 and the reverse primer targets exon 8 (product length =359 bp; the lower band detected in both samples is non-specific and was detected in two repeats of the experiment). For the comparison of *EEF2* expression in all samples product length is 173 bp.

Supplementary Table 1: List of guides used.

Use	Sequence
Lama2 KO in murine MuSCs - guide 1	CCGATTACGAATGCTATTGA
Lama2 KO in murine MuSCs - guide 2	CAGAGTCCCAGTATCAAGAA
Lama2 KO in murine MuSCs - guide 3	GATTGCAGATTCGGCACTGA
LAMA2 Ex3 KO in hiPSCs – guide 1	TCATCTTATCAAGAAAACAATGG
LAMA2 Ex3 KO in hiPSCs – guide 2	CTGTATGGTGCTATGAGACAAGG
LAMA2 Ex7 KO in hiPSCs – guide 1	CCTAGAGGCCTAGGAATCAAC
LAMA2 Ex7 KO in hiPSCs – guide 2	GACCAGTTGTCACTATTAGGCT

Supplementary Table 2: List of primers used.

Gene	Figure	Forward	Reverse
Lama2	1b	TGCCCTTTCTCACCCACCCTT	GTTGATGCGCTTGGGAC
Pax7	1b	GAGGTGACAGGAGGCAGAAG	AGCTGCCAGCAAGATGGTAT
Myog	1b	ACTCCCTTACGTCCATCGTG	CAGGACAGCCCCACTTAAAA
Gapdh	1b, S8e	ACCCAGAAGACTGTGGATGG	GGATGCAGGGATGATGTTCT
Lama2 exon 3	5b	AGCACCTTTCCAACAGGAGA	TGTGCTGTTGTGTTCCCTTC
TIDE sgRNA 1+2	S7b	AAGGCTGGTGGTCAGTGTTT	TCAGCATCGCTCCCAACTTT
TIDE sgRNA 3	S7b	AGGACCCGAGATGTACTGCA	TCTGTGGCCAGGGAGTCTAA
Itgb1	S8e	AGACTTCCGCATTGGCTTTG	GCTGGTGCAGTTTTGTTCAC
LAMA2 (control vs *LAMA2* Ex 3 KO)	S10	CGACCAATGCAACATGTGGAG	CTGCCACCAAGTGTTCTTTCCA
LAMA2 (control vs *LAMA2* Ex 7 KO)	S10	TTTCAGTTGGAGGGATGTGC	GGGTCTGAAGAAGCCATCAGT
EEF2	S10	TCAGCACACTGGCATAGAGG	GACATCACCAAGGGTGTGC

References

1. Ham AS*, et al.* Single-nuclei sequencing of skeletal muscle reveals subsynaptic-specific transcripts involved in neuromuscular junction maintenance. *Nature Communications* **16**, 2220 (2025).

2. De Micheli AJ*, et al.* Single-Cell Analysis of the Muscle Stem Cell Hierarchy Identifies Heterotypic Communication Signals Involved in Skeletal Muscle Regeneration. *Cell Rep* **30**, 3583-3595 e3585 (2020).

3. Murphy MM, Lawson JA, Mathew SJ, Hutcheson DA, Kardon G. Satellite cells, connective tissue fibroblasts and their interactions are crucial for muscle regeneration. *Development* **138**, 3625-3637 (2011).

4. Platt RJ*, et al.* CRISPR-Cas9 knockin mice for genome editing and cancer modeling. *Cell* **159**, 440-455 (2014).
