# Supplementary material for: Loss of cell-autonomously secreted laminin-α2 drives muscle stem cell dysfunction in LAMA2-related muscular dystrophy
Source: Nat Commun. 2025 Nov 27;16:10674. doi: 10.1038/s41467-025-65703-1 (PMC12661008; doi:10.1038/s41467-025-65703-1)
Supplement: Supplementary file 3 — Description of Additional Supplementary Files [file 41467_2025_65703_MOESM3_ESM.pdf]

Description of Additional Supplementary Files

File Name: Supplementary Data 1

Description: List of antibodies used.

File Name: Supplementary Movie 1

Description: MuSCs co-localize with laminin-α2 in the interstitial space and inside ghost fibers 4 days post-injury.
